# Intention Prediction for Active Upper-Limb Exoskeletons in Industrial Applications: A Systematic Literature Review

**DOI:** 10.3390/s25175225

**Published:** 2025-08-22

**Authors:** Dominik Hochreiter, Katharina Schmermbeck, Miguel Vazquez-Pufleau, Alois Ferscha

**Affiliations:** 1 Institute of Pervasive Computing, Johannes Kepler University, 4040 Linz, Austria; 2 Chair of Production Technology, Institute of Mechatronics, University of Innsbruck, 6020 Innsbruck, Austria

**Keywords:** intention prediction, active industrial exoskeletons, human–machine interaction, human–robot interaction, sensor modalities for exoskeletons, adaptive control in industrial applications

## Abstract

Intention prediction is essential for enabling intuitive and adaptive control in upper-limb exoskeletons, especially in dynamic industrial environments. However, the suitability of different cues, sensors, and computational models for real-world industrial applications remains unclear. This systematic review, conducted according to PRISMA guidelines, analyzes 29 studies published between 2007 and 2024 that investigate intention prediction in active exoskeletons. Most studies rely on motion capture (14) and electromyography (14) to estimate joint torque or trajectories, predicting from 450 ms before to 660 ms after motion onset. Approaches include model-based and model-free regression, as well as classification methods, but vary significantly in complexity, sensor setups, and evaluation procedures. Only a subset evaluates usability or support effectiveness, often under laboratory conditions with small, non-representative participant groups. Based on these insights, we outline recommendations for robust and adaptable intention prediction tailored to industrial task requirements. We propose four generalized support modes to guide sensor selection and control strategies in practical applications. Future research should leverage wearable sensors, integrate cognitive and contextual cues, and adopt transfer learning, federated learning, or LLM-based feedback mechanisms. Additionally, studies should prioritize real-world validation, diverse participant samples, and comprehensive evaluation metrics to support scalable, acceptable deployment of exoskeletons in industrial settings.

## 1. Introduction

**Figure 1:**
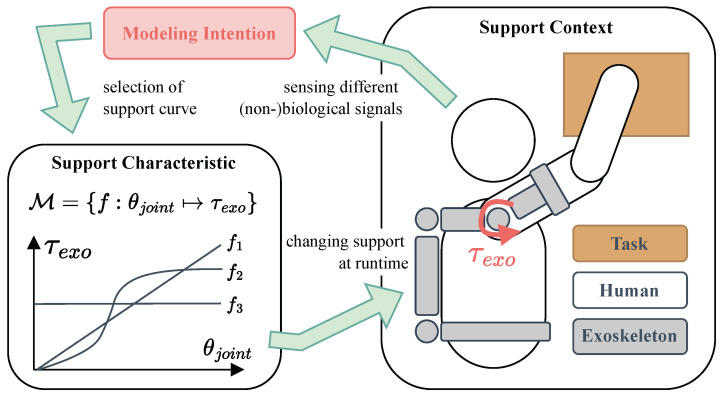
An active upper limb exoskeleton is defined by its mechanical properties and support characteristic, which is the set of available support curves. The effectiveness of the support depends on the requirements from the support context, which encompasses the exoskeleton, the human user, and the task. Modeling intention enables one to interpret the current requirements of the support context by analyzing various signals to select an appropriate support curve and adjust it during runtime. The figure illustrates this process using a torque-controlled shoulder exoskeleton as an example.

Exoskeletons are employed in industrial work settings to relieve specific body parts from physical stress by facilitating, adding, enabling, enhancing, or stabilizing movements [1]. Their primary objective is to lower the risk of work-related musculoskeletal disorders (WMSDs) [2,3], which are especially common in the upper limbs [4]. The effectiveness of an industrial exoskeleton depends on its ability to meet the dynamic requirements of the user, the task, and the environment. The support characteristic of an exoskeleton is described by the available support curves, which, for example, define the relationship between joint angle and the corresponding support force or torque (see Figure 1) [5].

While passive exoskeletons provide fixed support characteristics that cannot be changed during use, the actuation of active exoskeletons allows real-time adjustments. Systems that adapt their support automatically without user input are referred to as adaptive exoskeletons [6]. Developing such systems for upper-limb industrial use is challenging due to the need to accommodate frequent task and posture changes over long work periods [7,8]. Integrating intention prediction offers a promising approach to meet these challenges by enabling timely and appropriate support adjustments based on user behavior, somatic cues, or contextual information. This can reduce the user’s cognitive load and eliminate the need for disruptive manual tuning. In human–robot collaboration, intention prediction has already been applied to improve safety and task coordination [9,10,11]. Similarly, in rehabilitation exoskeletons, detecting residual motor function to guide support is critical for functionality [12,13]. However, despite this progress, intention prediction has received limited attention in industrial contexts, where exoskeletons remain largely passive and non-adaptive, often limiting their usability and effectiveness.

This review aims to provide the first structured overview of intention prediction methods relevant to active industrial exoskeletons and examine the transferability of rehabilitation-based approaches to industrial applications. Following the PRISMA guidelines for systematic reviews [14], we address two central research questions: *How is intention prediction implemented?* We address this by analyzing sensors modalities, applied methods, and models to obtain control outputs as well as prediction times. *Why is intention prediction implemented?* We address this by considering the desired support characteristics of systems and evaluation methodologies. The concise compilation identifies common strategies with their strengths and weaknesses and offers a foundation for guiding future research on intent-aware industrial exoskeletons.

## 2. Background

### 2.1. Active Upper-Limb Industrial Exoskeletons

Exoskeletons are wearable systems that provide support to specific body parts to add, facilitate, enable, enhance, or stabilize movements [1]. For industrial applications, they are employed to support human operators during physically demanding tasks, such as heavy material handling or overhead work. Although exoskeletons exist for various body parts, devices targeting the shoulders, back, and elbows are most prevalent in industrial contexts, as these areas are commonly affected by work-related musculoskeletal disorders (WMSDs) [4]. Empirical studies have shown that systems are able to reduce acute physical stress and strain in targeted body areas [2,3], thereby lowering risk factors associated with WMSDs. However, usability and acceptance remain limited due to a range of factors. In addition to issues related to comfort, social acceptance, and general usability [15,16], many industrial exoskeletons are developed for narrowly defined support contexts, tailored to a specific user, task, and environment [2,17]. While passive and active exoskeletons can be mechanically adapted to individual anthropometry prior to use, active systems incorporate actuators (e.g., electric or pneumatic) and control units that enable runtime adjustment of support forces or torques. These adjustments can be triggered manually or automatically via sensor data, using model-based or data-driven approaches [5]. Adaptive exoskeletons, i.e., those with automatic support adjustment, can enhance user experience and support effectiveness by minimizing unexpected or hindering support behavior during operation.

Recent research explores a range of approaches to detect changing support requirements in exoskeletons, leveraging environmental cues, kinematic or physiological signals from the human operator, or multimodal combinations [18]. The intended system behavior is inferred using models ranging from simple linear functions to complex biomechanical models, with control outputs generated by downstream system-level controllers. A comprehensive strategy for achieving adaptive support is to leverage users’ intentions, i.e., users’ future actions. While intention prediction is essential for the functionality of rehabilitation exoskeletons and has been intensively studied in recent years [13,19,20], its application in industrial exoskeletons remains in its infancy. In particular, it remains unclear which approaches from other exoskeleton application fields can be applied to real-world industrial contexts as they introduce additional requirements: short possible sensor setup times, higher movement velocities that demand low control latency times, the dynamics of human of movements from fine manipulation to high force compensation, and constant interaction with the external environment [7,8]. Moreover, as stated by Gantenbein et al. [18], today’s research on the effects of exoskeletons lacks standardized procedures, including clearly defined metrics and protocols. This lack of uniformity as well as publicly available datasets complicates the comparison and benchmarking of different approaches, impeding the systematic advancement of the field.

### 2.2. Intention Prediction

To enable proactive control of an exoskeleton, a computational model must effectively learn or predict the user’s intentions, dynamically adjusting support characteristics to meet evolving demands from the user, task, and environment. The distinction between intention recognition and prediction can be difficult due to their overlapping concepts and methods [21]. While intention recognition focuses on understanding current goals, prediction anticipates future intentions. In our structural analysis, we distinguish between biological and non-biological cues. This distinction is a key factor in our findings. Muscle activity, for example, is a biological cue that can be measured before movement. These cues act as input patterns that can be used to predict intentions. A variety of biological (somatic) cues can be observed, such as heart rate [22,23], skin conductance and muscle activity [24,25], gaze [26], photoplethysmography, electrodermal activity, brain oxygenation [27], and energy expenditure. Predicting emotional states through facial expressions, vocal cues, or physiological responses provides valuable insights [28,29], and cognitive context involves users’ mental state [30]. Non-biological cues consist of behavioral, social [31,32,33,34], contextual [35,36,37], and habitual patterns [38]. They include visible, intentional, and observable actions or motions, such as gestures [39,40], eye movements [26,41], or series of whole-body movements [42]. Analyzing movements can offer predictive cues about intentions and future actions [43,44].

Cue signals for kinematic data, for example, are monitored and captured using sensor technologies such as inertial measurement units (IMUs) or multiple inertial measurement units (M-IMUs). Applied forces can be measured using pressure sensors integrated into gloves or interfaces. Vision-based sensors, such as cameras, depth cameras, light detection and ranging (LiDAR) [45], or marker-based motion capture systems (MoCap), enable gesture or motion recognition and spatial mapping. Surface electromyography (sEMG) measures muscle activity to infer intended movement [46] or fatigue, while electroencephalography (EEG) records brain activity to detect cognitive processes. Galvanic skin response (GSR) sensors detect changes in skin conductance associated with emotional arousal. In vivo imaging techniques, like functional near-infrared spectroscopy (fNIRS) [27] and functional magnetic resonance imaging (fMRI), visualize brain activity to understand neural correlates of intention, while CMOS and low-cost semiconductor lasers detect vascular structures [18,47].

Computational and algorithmic methods use captured signals to infer intent. These signals are typically pre-processed and filtered before being fed into an algorithm to produce a discrete or continuous output. A distinction can be made between model-free, i.e., data-driven, and model-based approaches [11], the modalities used [18], and the prediction horizon. Implementations range from classical machine learning (SVM, decision trees, random forests) to deep neural network models [48]. For temporal inference of intentions or tasks, sequence models such as time series classification (TSC) can provide predictions, including recurrent neural network (RNN) models that capture the dynamics of sequences. RNN model architectures such as long short-term memory (LSTM), extended (xLSTM) [49], gated recurrent units (GRUs) and transformers use self-attention mechanisms to weigh the importance of different patterns in sequential applications.

## 3. Research Objectives and Methodology

Given these challenges and the fragmented state of current research, it is necessary to systematically evaluate existing intention prediction approaches across application domains to assess their relevance and applicability to industrial exoskeletons. To address this gap, the present review investigates intention prediction models used in upper-limb exoskeletons spanning rehabilitation, assistive, and industrial contexts, with the goal of identifying approaches suitable for real-world deployment in industrial upper-limb exoskeletons. The following section elaborates on the research questions we posed, the literature, and the PRISMA methodology [14] applied, including search strings, eligibility criteria, and analysis dimensions. This systematic review has not been registered.

### 3.1. Objectives and Dimensions

Two central research questions were formulated to explore the relevance and applicability of intention prediction research. First, we investigated *how* intention prediction is implemented, focusing on the types of sensors and computational methods applied, and the temporal characteristics of prediction. Secondly, we explored *why* researchers use intention prediction for exoskeletons, thereby analyzing target users, evaluation methods, and ways the system adapts based on the predicted intent. To address these research questions systematically during analysis, we defined seven analysis dimensions and formulated guiding sub-questions for each (e.g., A1: “What intention cues are used?”). These sub-questions were not additional research questions but served as a guideline during the review to ensure consistent extraction of relevant information. When a paper addressed or answered a sub-question, it was assigned to the corresponding analysis dimension, and relevant passages were highlighted for later synthesis. Table 1 provides an overview of the main research questions, their guiding sub-questions, and the associated analysis dimensions used to categorize and analyze the selected papers.

### 3.2. Methodology

This systematic literature review was conducted following the guidelines of the PRISMA methodology [14]. As a widely accepted method for systematic and reproducible reviews, we aimed to reduce the risk of missing data and incorporating biases into our analysis. We therefore organized our review along the recent PRIMSA guidelines [14]. You can find the data of the complete search process in the Supplementary Materials.

#### 3.2.1. Eligibility Criteria

**Figure 2:**
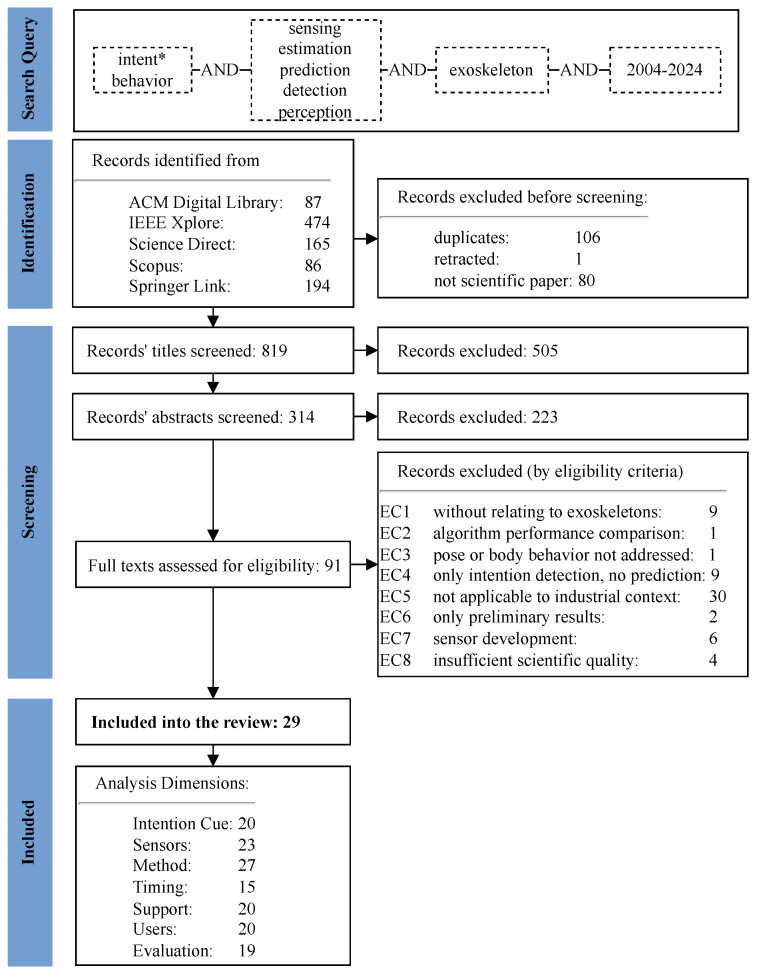
Detailed flow chart of PRISMA literature retrieval including the eligibility criteria (adapted from [14] to additionally show results of title and abstract screening). The top row displays the search terms and filters used in the databases. The asterisk (*) indicates that wildcard search was used, if possible, to find results including variations of the word.

The inclusion and exclusion criteria, shown in Figure 2, were chosen to ensure that the records’ content fit our research objectives. Prior to the assessment with eligibility criteria, records were excluded if they were not written in English or were workshop articles, from non-scientific literature, or duplicates.

The objective of this review was to identify sensors, models, and evaluation approaches for proactive control in exoskeletons that are transferable to industrial applications. Studies were excluded if their findings could not be directly applied to such control. This included work on intention prediction not designed for exoskeleton control (EC1), studies limited to sensor development (EC7) or algorithm benchmarking (EC2) without application context, and those presenting only preliminary results (EC6). Papers that did not use human poses as input (EC3) were excluded, as industrial exoskeleton support depends on user posture as this defines the load on the joint. Likewise, studies were excluded if they did not propose how their intention detection could be used for control or lacked timing information necessary to evaluate predictive feasibility (EC4). Applicability to industrial contexts (EC5) was a key criterion that was discussed among the three first authors. In cases of disagreement, studies were retained to avoid missing potentially relevant insights. We excluded reports presenting systems developed solely for rehabilitation purposes, where algorithms were designed for users with little or no movement capability. Moreover, studies presenting lower-limb exoskeletons were critically discussed, as these devices in industry are rarely used and typically limited to sitting or stair-carrying tasks. Exceptions were made when computational models or evaluation techniques appeared transferable, as in [50,51]. Finally, papers were excluded if their scientific quality was insufficient, for example, due to unclear argumentation, lack of methodological detail, or non-logical results.

#### 3.2.2. Databases and Search Strategy

All used databases and a general search query are listed in Figure 2 with the number of found records. If the options were available, records were filtered for English language, a time span from 2004 to the day of the search (3 February 2024) was implemented, and a search excluding words in references was selected. The search string, which was adapted to each database, was developed and discussed.

#### 3.2.3. Selection Process

The screening of titles, abstracts, and full texts was conducted by the first three authors. During the title and abstract screening, each author individually decided if the record met the criteria and unclear records were discussed. The full text screening was performed by two authors independently; exclusion criteria were recorded and subsequently discussed. This resulted in 29 relevant records published from 2007 to 2024.

#### 3.2.4. Data Collection and Synthesis

The data extraction was organized by analyzing the final paper selection, considering seven comparison dimensions, which were assigned to a responsible author. Table 1 gives an overview on the dimensions and their relation to the research questions, while Table 2 lists all resulting records and the respective analysis dimensions.

Due to substantial heterogeneity within our dimensions of analysis, a quantitative meta-analysis was not feasible. The included studies employed diverse model approaches (Figures 4 and 5) and only partially overlapping outcome measures (see Appendix A.2, Table A2), while several lacked extractable numerical results or were purely descriptive or review papers. Under these conditions, any statistical pooling would have risked producing misleading or non-interpretable results. In line with the PRISMA 2020 guidance for systematic reviews without synthesis [14], we therefore applied a narrative synthesis and descriptive benchmarking approach, grouping comparable methods to facilitate cross-study comparison while preserving the diversity of approaches in this field.

## 4. Results

**Figure 3:**
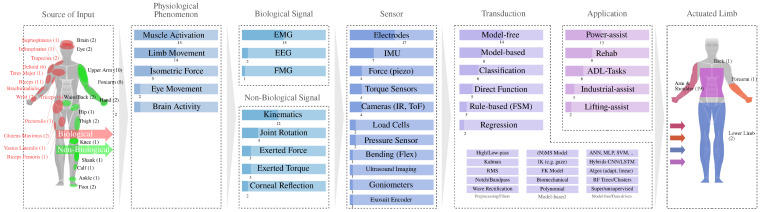
An overview of the distribution of methods included in this review, giving an indication of the approaches used to predict intention and control of an exoskeleton. The numbers represent the number of publications we found that used, applied, or addressed one of the items listed in each category. From left to right, this chain shows the process from the human body sensor source to the actuated limb, with all different intermediate steps covered in this review.

This section summarizes the results of the selected publications, shown in Table 2 along with the dimensions of our analysis. Figure 3 shows an integrated view of the distribution of methods for the entire intention prediction process, from the source of the input to the supported limb.

### 4.1. Intention Cues

User intent in human motor control involves the central nervous system (CNS), peripheral nervous system (PNS), musculoskeletal system, and environmental interactions, each with measurable state variables reflecting intention [62]. These variables are interconnected; for example, the PNS sends sensory data to the CNS, which plans motion and sends commands back.

The existing reviews and collections included in this review [11,18,62,74] presented other existing definitions and classifications of intention prediction cues. The Gantenbein et al. review [18] addressed techniques for recognizing and estimating intention in the analysis of usability, daily life applications, and user evaluation studies. For this purpose, they adapted the classification scheme of [76] to measure the distribution of input signal source, physiological phenomenon, signal to observer, and sensor technology among the selected studies. In [74], we found a discriminative criterion for sEMG-based motion intention recognition methods, which could be divided into two groups: musculoskeletal model- and machine learning-based. Similarly, for EMG-based continuous motion prediction models, we found a distinction between model-based and model-free approaches [11]. The review by [62] structured the communication of intent from user to robot into identification, measurement, and interpretation.

We organized the cues and selected methods in terms of periodicity, as shown in Figure 6, and sorted them according to their use. Intention cues were either measured for (i) intention and activity prediction, (ii) activity and movement onset and offset detection, or (iii) intention and activity classification.

#### 4.1.1. Intention and Activity Prediction

We found papers that used eye movements as input [66], which assumed that patients always fixated their gaze approximately on the target of a reaching movement. In terms of motion prediction, biological signals and muscle activity measured with EMG [11,68] could be used for acceleration and velocity prediction, joint angle and position prediction, and joint torque prediction [53]. Kinematic data and segmentation techniques, with IMU data, could be used to predict a worker’s intended motion. They required a minimum observation window for proactive control, such as 200 ms [72].

#### 4.1.2. Detect Onset and Offset of Activity

The work of Lotti et al. [63] presented a short-term, muscle-based intention estimator. The EMG-driven modeling was connected to the low-level controller of the exosuit. A subsequent comparative study [64] used a mechanical intrinsic controller, which was slower to react, but the exclusion of biosignals from the control framework made the reference torque more stable.

A study on short-term intention estimation in realistic overhead work tasks [75] evaluated the performance of kinematic control and EMG control. They used the user’s reaction time to the given cues as the reference for evaluating the controller’s reaction time in all trials. The onset response of the EMG controller was on average 0.17 s faster than that of the kinematics controller. The study by Heo et al. [57] used sEMG sensors to monitor muscle activities for onset estimation for two types of activities, squatting and bending. It compared the estimation latency between sEMG sensors and kinematic sensors, i.e., IMU-based motion capture. Generally, EMG assistance was triggered earlier than kinematic assistance. The latency even increased with the weight lifted. This was because heavier external loads reduced the lifting speed, which caused a delay in the onset time of kinematic-based lift detection. In their study [67], a continuation to [66], Novak and Riener presented EMG and torque sensors for onset detection, with an offline trained prediction model.

Intention to stand up can be observed at the ankle, knee, and hip angles using goniometers and pressure sensors placed on the sole of the foot [50]. The work of [55] involved developing a human–machine interface based on a wrist-level force sensor; minimal force detection in the six directions in 3D space represented the input to obtain the desired incremental movement of the end-effector in space.

#### 4.1.3. Intention and Activity Classification

The work of Irastorza-Landa et al. [59] developed a classification system for predicting movement intention and intended direction in point-to-point horizontal-plane reaching movements based on the EMG activity of five upper arm muscles of healthy subjects. A classification accuracy of over 70% was achieved in offline evaluations. The kinematic approach of [72] used IMU motion data. A movement intention and sequence prediction model was verified by training an MLP model and predicting the motion sequence of new data. Bandara et al. [52] proposed a non-invasive BCI approach to study the dynamic features of EEG signals occurring during ADL tasks. In an offline analysis, the activated brain regions and frequency ranges were identified for each of the intended user movements. This was carried out for individual users as well as for a common model for all users during their study. The identified signals were then used in real time as input to neural networks and SVM-based classifiers to predict intended movements. The work [67] on eye movement reported on target prediction using gaze position alone. In an analysis, they found that context alone could predict the target area about 50–60% of the time.

Significantly improving the prediction and classification of human intentions requires the integration of biological signals, such as EMG and EEG, alongside sensing technologies and ML models (see Section 4.3). Although kinematic approaches are slower than EMG-based systems, they provide valuable and robust additional information for understanding movement dynamics.

### 4.2. Sensors and Signals

To explore how intention cues are captured and the signals used in predictive models, we analyzed the sensors and physiological signals across studies, distinguishing biological signals originating from neuromuscular or cognitive activation from non-biological signals reflecting external or motion-related traits. This separation streamlines the categorization of appropriate sensors for specific applications by taking factors such as precision, response time, and stability into account. It also eliminates the added complexity of overlapping tiers. Further, this distinction aligns with the practical challenges and requirements of industrial exoskeletons, such as setup time, calibration needs, and environmental robustness.

#### 4.2.1. Biological Signals

Biological signals have the advantage of detecting neuromuscular activation before actual movement onset [68], as shown in Figure 6. There are different signal types, such as muscle activity, eye movement, and brain activity, that are commonly measured using electrodes, with EMG and EEG being the primary modalities.

Portable dry-electrode EMG devices improve user comfort and reduce setup time but often suffer from lower signal-to-noise ratios compared to wet electrodes [11]. Wearable devices like the Myo-Armband with multiple dry electrodes have been successfully used to estimate joint angles and angular velocities of shoulder and elbow [69] and movement classes, i.e., rest flexion, extension, and external load [71]. The majority of studies, however, use wet bipolar Ag/AgCl electrodes. As shown in Table A1, biceps and triceps brachii are frequently used for estimating elbow torque, while more complex estimations incorporate deltoid or back muscles [53,68]. When applying data-driven classification models, studies often use up to 12 EMG channels. Chen et al. [54], for instance, classified arm motions such as drinking or reaching. Simpler approaches detect lifting motion using leg muscles [57] or arm muscles [75], motion onset [50,67], or reaching directions [59].

In addition to myoelectric signals, one study used EEG to infer intended arm movement in the 3D space from brain activity [52] or employed eye tracking to identify reaching goals based on gaze direction [66,67].

In summary, EMG can detect neuromuscular activation before movement onset, especially from the biceps and triceps, and is the most commonly used technique to estimate torque, movement classes, and joint kinematics. This method is advantageous for its fast response times. Although portable dry-electrode devices have improved usability, wet electrodes remain dominant. However, both types of electrodes lack environmental robustness and require calibration. Studies often use multiple EMG channels or combine modalities, such as EEG and eye tracking, to infer more complex movement intentions [66]. This can contribute to stability and precision.

#### 4.2.2. Non-Biological Signals

Kinematic and dynamic sensors are commonly used to capture motion-related information such as acceleration, velocity, orientation, position, and joint or limb forces. IMUs, which typically combine accelerometers, gyroscopes, and magnetometers, are widely used to estimate joint angles or body segment orientations in the 3D space. All reviewed studies calculated global angles and angular velocities either through the IMU’s internal algorithms [51,57,64,70,72,75] or external methods such as the Mahony algorithm, which is robust to sensor orientation [65].

Some approaches leverage sensors already embedded in exoskeletons. Lotti et al. [63] used the electrical motor encoder to extract elbow joint positions, while Dinh et al. [7] integrated a flex sensor to monitor elbow bending. For torque prediction, force sensors or load cells are frequently embedded at the user–exoskeleton interface to detect external loads and movement directions [53,55,58,60,66,67]. Two studies employed vision-based systems using IR depth sensors (Kinect® v2) to estimate user poses [56,61].

As observed in the reviewed studies, IMUs offer high flexibility in placement and can transmit data via wireless protocols, making them well-suited for mobile setups and various body segments. In contrast, force sensors and embedded motor sensors like encoders are structurally integrated into the system and require wired connections, but they provide highly precise, low-drift measurements that are advantageous for stable and accurate angle and interaction force determination.

### 4.3. Computation and Models

Researchers apply classification or regression models to predict user movements or joint torques as discrete or continuous outputs. For continuous prediction, methods are often categorized into model-based and model-free approaches [11,74]. Model-based techniques rely on biomechanical or kinematic knowledge to analytically link sensor signals with joint angles or torques. In contrast, model-free approaches use data-driven techniques, such as neural networks (NNs), to learn input–output relationships.

#### 4.3.1. Classification

**Figure 4:**
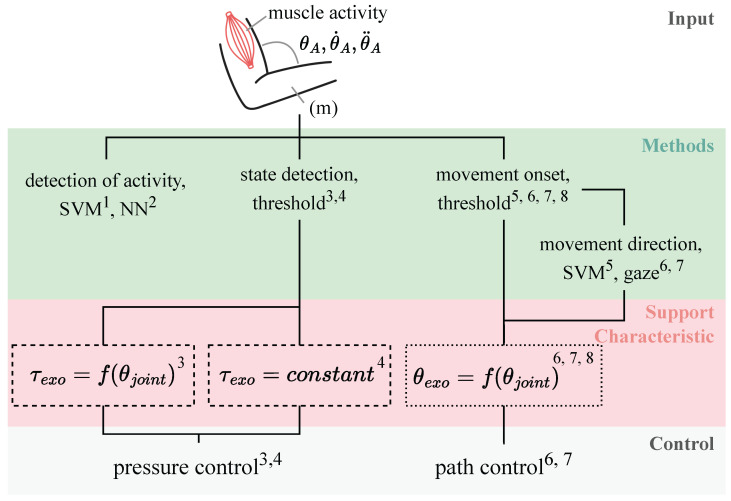
Classification-based intention prediction typically uses threshold methods to detect activities (^1^ [52], ^2^ [50]), activity states (^3^ [55], ^4^ [75]) or motion onset (^5^ [57], ^6^ [64], ^7^ [65], ^8^ [71]). The support characteristic is then modeled as a constant or a function of joint position (dashed = torque, dotted = trajectory). Line endings within the stream indicate unspecified support characteristics.

As shown in Figure 4, eight of the analyzed research papers used classification to detect user intent or activity. Zhou et al. [75] implemented a binary, threshold-based classifier to distinguish between support and non-support states. They found that IMU-based control achieved higher accuracy than EMG-based control. Other works combined binary classifiers with movement direction prediction. Irastorza-Landa et al. [59] used an EMG-based support vector machine (SVM) to detect onset and reaching direction. Novak and Riener [66,67] initially relied on velocity thresholds and later switched to a hybrid EMG/torque-based threshold method, achieving robust early detection (35.4 ms before motion, with 3.9% false positives).

Other studies implemented multi-class classification to recognize specific activities. Heo et al. [57] implemented a threshold-based finite state machine to detect lifting phases (e.g., grasping, lifting), supporting users only during the lift. Bandara et al. [52] and Chen et al. [54] classified activities of daily living, like drinking or reaching, using EEG and EMG inputs, respectively. Both compared classifiers and found that SVMs outperformed NNs in accuracy. However, Bandara et al. reported faster detection with NNs (300 ms vs. 600 ms on average), while Chen et al. noted that NNs require significantly more training data, which limits performance on smaller datasets.

Binary classification enables fast, low-complexity detection for distinct movement situations, while multi-class classification offers broader situation coverage but demands more data and careful algorithm selection based on the requirements for accuracy, latency, and computational costs.

#### 4.3.2. Model-Based Regression

**Figure 5:**
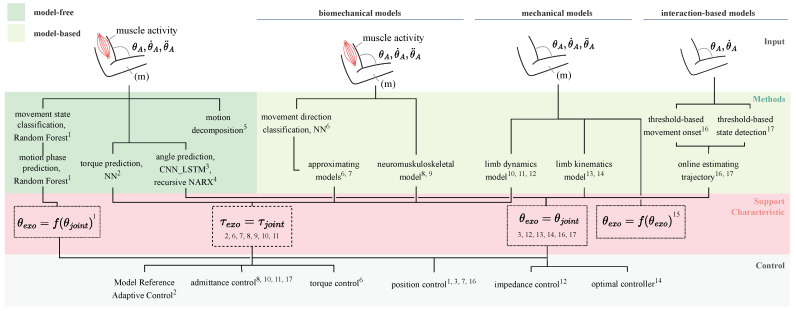
Regression-based intention prediction is implemented based on either model-free approaches (^1^ [51], ^2^ [70], ^3^ [68], ^4^ [69], ^5^ [72]) or models-based approaches including biomechanical (^6^ [71], ^7^ [65], ^8^ [53], ^9^ [63]), mechanical (^10^ [64], ^11^ [7], ^12^ [60], ^13^ [61], ^14^ [56], ^15^ [73]), or interaction-based models (^16^ [55], ^17^ [58]). The support characteristic is then modeled as a constant or a function of joint position (dashed = torque, dotted = trajectory). Line endings within the stream indicate unspecified support characteristics.

Researchers use model-based approaches to continuously estimate joint torques or motion trajectories (see Figure 5). These can be grouped into three categories: biomechanical models, which simulate neuromusculoskeletal dynamics; mechanical models, which rely on kinematic or dynamic, i.e., integrating forces and equations of motion; and interaction-based models, which infer intent from user–exoskeleton interface forces without modeling human biomechanics.

Biomechanical models use measured muscle activity and anatomical data to estimate joint torques. Buongiorno et al. [53] and Lotti et al. [63] estimated muscle forces via Hill-type models and OpenSim-based simulations. Lotti et al. calibrated the model through subject-specific maximum voluntary contraction (MVC) and movement trials, while Buongiorno et al. applied optimization methods. Simpler models like those by Lu et al. [65] and Treussart et al. [71] mapped EMG signals to torque using exponential functions with acceptable error levels.

Mechanical models apply physical equations to predict torque or trajectories. Lotti et al. [64] estimated elbow torque using inverse dynamics, integrating IMU data and users’ anthropometrics. Dinh et al. [7] derived torque from measured cable tension and the arm’s modeled potential and kinetic energies. Khan et al. [60] combined a dynamic arm model with an NN to adapt to subject-specific and time-varying dynamics. Vision-based methods by Liao et al. [61] and Gao et al. [56] use kinematic models.

Interaction-based models extract user intent directly from forces at the physical human–exoskeleton interface to classify motion states and directions [58] or treat force readings as joystick-like inputs [55].

Biomechanical and mechanical models offer physically grounded and accurate predictions of torques but require detailed modeling of both the user and system. This makes the setup more complex and calibration procedures are inevitable. Interaction-based models are simpler and rely on embedded sensors but are limited to the exoskeleton’s structure.

#### 4.3.3. Model-Free Regression

Model-free regression approaches use machine learning to learn the relationship between sensor inputs and torque or trajectory outputs based on training data. Sedighi et al. [68] combined a CNN and LSTM to predict shoulder and elbow trajectories from EMG signals and joint angles. Sun et al. [69] employed EMG data and a NARX network with an unscented Kalman filter to estimate joint angles. Woo et al. [72] segmented lower-limb movements using k-means clustering on IMU data and applied an NN to generalize predictions across subjects. Toro-Ossaba et al. [70] predicted elbow torque from recent biceps and triceps EMG using an NN. Zhang et al. [51] applied random forest models for locomotion mode and gait phase classification based on hip and shank kinematics. While model-free methods do not require prior knowledge of the system’s dynamics or biomechanics, their performance depends on data quality and quantity and they require additional effort to generalize beyond the trained conditions.

Classification and regression methods each offer advantages depending on the requirements, such as available sensors, modeling capabilities, and application context. Classification is particularly effective for recognizing discrete motion states or transitions, such as detecting movement onset or distinguishing between lifting and standing. Threshold-based approaches enable simple, fast, and robust implementations. Regression methods, on the other hand, allow for continuous prediction of joint angles or torques and are better suited if support characteristics must change continuously. Model-based regression requires detailed biomechanical or mechanical modeling and subject-specific calibration, while model-free regression relies mainly on training data, offering greater flexibility but demanding careful data preparation and generalization strategies.

### 4.4. Time

**Figure 6:**
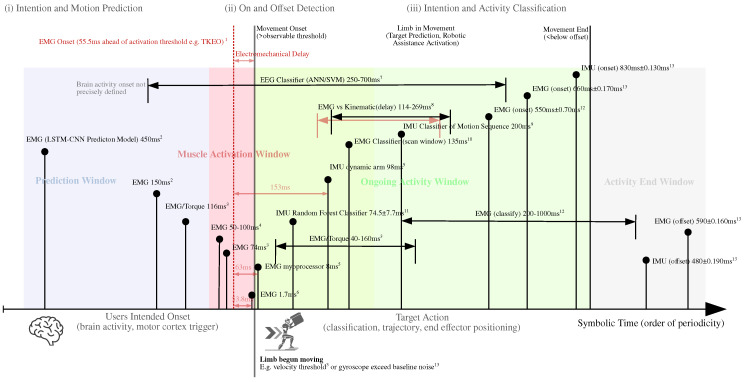
Overview of symbolic time windows to contextualize intention prediction in existing studies (^1^ [77], ^2^ [68], ^3^ [67], ^4^ [11], ^5^ [64], ^6^ [63], ^7^ [52], ^8^ [57], ^9^ [72], ^10^ [54], ^11^ [51], ^12^ [59], ^13^ [75]). The left vertical line indicates movement onset, serving as a key reference to distinguish between prediction of movement onset (left of the line) and classification and regression of movements (right of the line). Movement onset is defined as the point at which a measurable, predefined threshold is crossed (e.g., velocity or muscle activation). Some entries, such as EEG-based approaches, are placed approximately due to imprecise onset definitions in the original studies. To improve comparability between studies, EMG-based onset timings were adjusted by subtracting the electromechanical delay (indicated by red arrows), accounting for the lag between muscle activation and mechanical movement.

Timing the intention detection and system reaction plays an important role for an effective and natural support. As shown in Figure 6, different aspects of time must be considered, such as the computational delay, the mechanical system delay, and the chosen prediction and analysis windows. Moreover, time components are intertwined with the chosen prediction method. When applying classification methods, for example, the detection time of activity onset is critical for the support effect [57].

#### 4.4.1. System Response Time

The system response time is the total duration from the initial sensor input signal to the resulting measurable torque or force applied to the user’s limb. Time delays can be due to different causes including sensor type, algorithms, filtering, and hardware [51]. Using biological signals, such as brain or muscle activity, allows one to detect motion prior to its actual execution. This *electromechanical delay*, also shown in Figure 6, between the EMG activity and a mechanically measurable muscle contraction is quantified in the literature as being between 30 and 150 ms [74,77], depending on the physiological state, sensor type, and threshold criterion. Sedighi et al. measured the EMG activity an average of 150 ms before the movement onset [68]. Brain activity, on the other hand, can be measured even earlier before muscle contraction, but is prone to false positives [78]. Multiple authors compared the difference in the computational delay of biomechanical and mechanical models predicting torque, with biomechanical approaches being significantly faster due to earlier signals from biological than non-biological sensors [57,64,75].

Researchers present different approaches to reduce computational and inherent system delays. Zhang et al. applied a time-delay compensation parameter to account for filter and hardware delays, which reduced their system response time by around 43 ms, with a total delay of 0.745 ± 0.077 s. Sedighi et al. predicted the user’s arm position 450 ms into the future to compensate for system delays [68]. Liao et al. reduced their system response delay by 38% to 0.5 s for fast motions by applying a Kalman filter [61]. Riener an Novak optimized their response time by learning the onset threshold of the EMG signals for each user separately [67].

#### 4.4.2. Analysis Times

Researchers analyze sensor signals at different frequencies and time spans (Figure 6). For models with continuous output, the sampling frequency determines the analysis time. EEG as well as EMG signals are commonly sampled at around 1 kHz and subsequently filtered [11,50,52,53,57,63,65,71,75]. Dinh et al. and Lotti et al. also sampled their motor control, IMU, and position signals at 1 kHz [7,63,64]. Woo et al. used IMUs sampling on 64 Hz [72]. Vision-based methods, such as Vicon, Kinect, or eye-tracking, have considerably slow sampling rates of 330 Hz [73], 30 Hz [61], and 60 Hz [66], respectively. For classification or model-free regression methods, a sliding window approach is used for feature extraction with an overlap [11]. Chen et al. used a sliding window of 540 ms with 81 ms of overlap to extract features from EMG signals for training and a 135 ms window during online classification [54]. Irastorza et al. compared sliding window length for feature extraction of EMG and found that 200 ms or 500 ms windows did not affect classification accuracy, but a short window allowed faster system response [59]. Similarly, Toro-Ossaba et al. used a sliding window of around 200 ms with 50% overlap [70] and Zhang et al. used a sliding window of around 300 ms but with 96% overlap [51]. Sedighi et al. used a 30 ms sliding window with 2 ms steps for EMG and joint angle for a CNN-LSTM model [68].

### 4.5. Support Characteristic

The support characteristic defines the type and amount of support an exoskeleton provides and is critical to both its effectiveness and usability. It is typically described by the targeted joint(s), the control strategy, and the resulting force or torque profile applied to the human limb [5]. As shown in Figure 4 and Figure 5, support strategies are broadly categorized into classification or regression approaches to derive either a target torque or a desired trajectory. These strategies differ in implementation complexity, resemblance to natural human movement, and adaptability to external factors such as load variations.

Torque-based control is often implemented for a single joint, most commonly the elbow, shoulder, or hip. Notable exceptions include Treussart et al. [71] and Buongiorno et al. [53], who simultaneously support both elbow and shoulder. Biomechanical models estimate joint torque using muscle activity signals, aiming for a direct equivalence between biological and assistive torques ($\tau_{joint}=\tau_{exo}$). These models typically employ admittance control, where the exoskeleton’s response depends on measured or calculated forces. Lotti et al. [63] and Buongiorno et al. [53] implemented such systems using detailed neuromusculoskeletal models and subject-specific calibration. Simpler variants using approximated exponential mappings offer reduced accuracy but benefit from faster computation [65,70,71]. Lu et al. fed torque predictions into a closed-loop position control system [65], while Treussart et al. applied a Dead Zone Integral corrector to refine torque control [71]. Mechanical models apply classical dynamic equations to estimate support torques. These are typically executed using admittance control, as seen in Dinh et al. [7] and Lotti et al. [64]. In contrast, interaction-based models use simpler mappings between body kinematics or interface forces and control output. These are often realized via closed-loop pressure control in pneumatic systems, providing gravity compensation or constant support torques [57,75].

In trajectory-following strategies, the goal is to ensure that the exoskeleton follows the user’s joint motion ($\theta_{joint}=\theta_{exo}$), often enhanced by gravity compensation. Biomechanical models such as the CNN-LSTM network by Sedighi et al. [68] infers joint trajectories from EMG signals and implement position control, enabling automatic adjustment to varying loads. Mechanical approaches incorporate limb dynamics into impedance control architectures, sometimes augmented with learning algorithms (e.g., Extreme Learning Machines) to forecast future states [60]. Simpler strategies based on vision-based motion tracking directly map observed limb positions to control inputs without estimating interaction forces [56,61]. Researchers most commonly employ interaction-based trajectory controllers, which either estimate movement paths online or follow predefined trajectories. Gandolla et al. [55] estimated the motion trajectory from end-effector force vectors and applied it within a gravity-compensated position control scheme. Huo et al. [58] classified movement modes and used Kalman filter-based admittance control based on link-specific force sensors. Other systems rely on predefined trajectories derived from optimality criteria such as energy minimization [73], minimum jerk [66], or straight-line motion [67], executed through standard position control. Zhang et al. [51] supported hip motion using fixed trajectories tailored to walking scenarios classified in real time.

As shown in the reviewed studies, the choice of control scheme depends on whether the exoskeleton is designed to achieve a certain torque or trajectory. Torque-based approaches typically use admittance control to adjust support based on user-generated forces, particularly in biomechanical and mechanical models. Some also apply closed-loop pressure or position control, in one case combined with a corrective control scheme. In contrast, trajectory-based methods rely mainly on position control to follow user motion, with impedance or admittance control used when accounting for interaction forces or dynamic predictions.

### 4.6. Users and Evaluation

The majority of reviewed studies developed intention prediction for rehabilitation or assistive exoskeletons, with only 20% targeting an industrial application. Two publications [51,53] did not specify the application context. Evaluations were exclusively conducted in laboratory settings with mostly healthy participants (1–10 per study, mean = 6 participants; 72% male; mean age = 27.2 years). Only Gandolla et al. [55] and Zabaleta et al. [50] conducted preliminary tests with one impaired user. No field studies were reported.

Study objectives generally fell into two categories: model evaluation or assessment of usability and effectiveness. Classification algorithms were evaluated using classification accuracy on recorded test datasets, as seen in Table A2. Only Riener and Novak [67] reported false positive errors on movement intention detection. Lotti et al. [64] compared mechanical and biomechanical models based on response and settling times after perturbations, showing faster computation but reduced stability for the biomechanical model. Continuous regression models were evaluated by comparing predicted and measured trajectories or torques. Most reported quantitative error metrics, i.e., root mean square error (RMSE) or coefficient of determination R^2^, though some provided only qualitative assessments [55,58,61]. Protocols included single-plane joint motions (e.g., elbow flexion [7,63]) or functional tasks (e.g., drinking [60], object manipulation [64], activities of daily living [73]). Only interaction-based models were tested for industrial-like tasks, such as overhead work and lifting [57,75].

Support effectiveness was primarily assessed via changes in muscle activity. All elbow supporting exoskeletons led to reductions in bicep activation [7,63,64,65], with Lotti et al. also noting reduced triceps, brachioradialis, and deltoid activity. Treussart et al. [71] found decreased activation in the biceps, deltoid, and erector spinae during load lifting, but no significant changes in other muscles. Heo et al. [57] observed reduced activity in lumbar and leg muscles during squat lifting with their back exoskeleton, but no significant changes in the gluteus maximus or biceps. Their EMG-triggered system also reduced fatigue of the longissimus thoracis during stoop lifting, measured by the decreasing mean frequency of the sEMG power spectrum. Mechanical work was evaluated in two studies. Lotti et al. reported lower user effort with their biomechanical model [64], while Heo et al. identified an optimal control delay (0–100 ms), balancing mechanical support and muscle activity reduction [57]. Lotti et al. [64] assessed kinematic changes, finding no significant alterations in range of motion or peak joint velocities of elbow and shoulder flexion/extension. Zhang et al. [51] reported a significantly higher maximum thigh angle and peak velocity during support.

Four studies included user feedback through interviews or questionnaires [56,58,66,71]. Participants generally reported good support, especially for the arms and shoulders, but noted reduced efficiency compared to traditional assistance. Perceptions of comfort, fatigue, and control were also discussed. However, Huo et al. and Novak and Riener did not disclose the questionnaire content [58,66].

Evaluations remained limited to lab settings with small numbers of healthy participants who did not constitute target user groups. While classification and regression models were assessed using standard performance metrics, support effectiveness was mainly analyzed through EMG-based muscle activity, with some analyses of fatigue, mechanical work, or kinematics. Only a minority of studies incorporated subjective user feedback, and standardized usability evaluation protocols were largely absent.

## 5. Discussion

This systematic review provides a comprehensive overview of studies evaluating active exoskeletons’ control strategies and the inclusion of prediction methods that could be used in industrial applications. This work builds upon and extends existing reviews in the field, such as those by [11,18,62,74], with a particular emphasis on methods for industrial contexts. By narrowing its focus, it aims to address research gaps and provides insights into the development and evaluation of industrial exoskeletons. The choice of search terms and eligibility criteria influences the identified papers during the search process. Our chosen search criteria (Figure 2) may have excluded other relevant terms, such as “motion prediction”. The results were biased by our search focus, which favored work applicable to an industrial context. This included multimodal control strategies, exoskeleton actuation, task execution, and sensor perception. However, to ensure that all relevant research was considered, full texts were evaluated by the three first authors separately, especially the applicability to industrial contexts, and disagreements were discussed.

The possibility of predicting intentions must be critically assessed. The questions raised by Boncheck-Dokow and Kaminka [79] highlight the limitations of using computational methods for this purpose: *How is intention prediction possible when only a failed sequence of actions is demonstrated? How is intention prediction possible when the actions are performed on novel objects, about which the observer seemingly has no prior knowledge?* This challenges Leibniz’s “Calculemus”. We humans can only estimate what might happen on the basis of observations and rational assumptions. The latter is only partially true. According to psychologist Daniel Kahneman, people prefer individual experiences over probability judgements [80]. The process of predicting the intentions of an agent, whether human or machine, is defined by its stages and indicators. Humans can make associations and hypotheses about possible reasons and goals behind actions or behavior, such as the process of “mind-reading” [81]. This, in turn, can be used to predict future outcomes, such as sequences of actions, and provide a blueprint for computational models. However, this is only a best estimate.

### 5.1. Intention Cues

The collected work shows a number of different cues that support an intention-based control strategy of active exoskeletons. As shown in Figure 6, the cues analyzed in the review range from short-term estimation based on muscle activity to predict the start of a movement to the use of classifiers that can take several hundred milliseconds to predict the actual activity. The somatic cues, such as the most commonly used EMG-based muscle activity, tend to work fast for prediction strategies, while behavioral or situation-context cues, such as signals derived for movements, tend to be slower because they require a longer observation window for online prediction [63]. For behavioral cues, such as kinematic signals measured by IMUs or cameras, an observable activity must have already occurred, but this method is less prone to errors and results in stabler control signals.

Implementing intention prediction in upper-limb exoskeletons involves processing intention cues derived from biological and non-biological signals. Biological signals such as muscle activity (EMG), brain activity (EEG), and somatic indicators are valuable because they reflect neuromuscular activation before physical movement occurs, enabling the early detection of user intent [11,79]. For instance, EMG-based methods can predict motion onset up to 450 ms in advance, thereby minimizing response latency in dynamic environments [68,70]. Non-biological signals, including kinematic data, gaze patterns, and environmental interactions, can be captured using technologies such as IMUs, motion capture systems, and gaze trackers [18,56]. Although these signals lack the temporal immediacy of biological cues, they provide stable and robust data, thereby enhancing system reliability [70,71]. Integrating these cues requires sensor technologies and computational models. Biological signals are captured using EMG and EEG sensors, while non-biological signals rely on IMUs and cameras [6,56]. The data is then processed using machine learning models ranging from classical approaches such as SVMs to advanced deep learning techniques like CNNs and LSTMs [7,63]. Depending on application needs, these models classify discrete states or predict continuous variables such as joint torque [68,70].

A key challenge lies in balancing the sensitivity of biological signals with the stability of non-biological ones. Multimodal approaches combining both types of signals can enhance accuracy and robustness, ensuring effective responses to diverse tasks [65,70]. However, variability in user behavior and the dynamic nature of industrial tasks necessitate more adaptable models. Future research should therefore focus on integrating multimodal cues, developing standardized evaluation protocols, and validating systems in real-world industrial settings, with the aim of improving usability and effectiveness [56].

### 5.2. Sensors and Signals

Sensors can improve exoskeleton control by capturing user intent signals and, with proper models, predicting pose and loads to reduce musculoskeletal stress.

Electromyography (EMG) was the most widely used biological signal in the reviewed studies. Its main advantage is the ability to implicitly capture changes in external load. However, EMG faces several practical challenges that limit its industrial applicability. EMG signals are highly user-specific, non-stationary, and sensitive to electrode placement and skin condition, making long-term reliability problematic [11]. Calibration, such as MVC scaling, is typically required for threshold-based methods [63,75], increasing setup time. Sweat-related degradation further complicates prolonged or high-mobility use [57]. Dry EMG sensors present a viable alternative, with comparable signal quality and faster setup than traditional gel electrodes [71,82].

EMG signals exhibit significant variability due to individual differences and environmental factors, which can differ not only between users but also across experimental sessions for the same individual [11,57]. The commonly employed normalization method, MVC, serves as a reference standard but often yields inconsistent maximum values across sessions, further complicating its application. These challenges highlight the necessity of more robust and adaptive normalization techniques to improve the reliability and reproducibility of EMG-based systems in industrial settings. One possible step toward this goal is real-time normalization or drift compensation, which uses adaptive Kalman or moving average filters to track trends and normalize EMG relative to recent activity levels. Dynamic, task-based scaling, which calibrates based on functional tasks, could produce repeatable results for control inputs. Other possible steps include the inclusion of musculoskeletal models tailored to the subject’s anatomy or the deployment of subject-specific training to learn EMG patterns without relying on explicit MVC scaling.

Recent developments in textile-embedded EMG, e.g., integration into underwear or compression garments, promise further improvements in usability and setup time, paving the way for real-world deployment [83,84,85]. Beyond EMG, other textile-based sensors measuring strain, respiration, or load handling may broaden intent inference and enhance multimodal systems [86,87,88].

Non-biological sensors, such as force, position, and velocity sensors, offer robust, long-term performance without the variability or degradation issues typical of biosignals. For example, force-based inverse dynamics models can estimate joint torques using mechanical signals alone, showing stable and reliable performance over time [64]. Such models also avoid issues like signal loss or sweat interference and are often simpler to implement. However, they may react more slowly and fail to account for user intent under changing external dynamics; additionally, model set-up can be time-intensive. Interaction forces can also support gaze-based interfaces by resolving selection ambiguity (e.g., the “Midas touch” problem) [66]. Additionally, context-aware sensing, such as BLE-based or UWB-based proximity or tool recognition, may be used to infer the weight or type of object the user is interacting with [89,90], further enhancing prediction accuracy.

Biological signals such as EMG enable fast and intuitive intent detection but require user-specific calibration and are sensitive to environmental conditions. Recent advances in dry and textile-integrated EMG sensors offer promising improvements in usability and robustness, increasing their suitability for industrial applications. In contrast, non-biological sensors provide greater long-term reliability and ease of integration but may compromise responsiveness to user intent.

When evaluating sensor technologies for industrial exoskeletons under conditions such as moisture, mechanical friction, and long-term alignment shifts, non-biological sensors such as IMUs and force sensors emerge as the most robust and reliable options [64]. IMUs can be placed in various locations, transmit data wirelessly, and possibly require filtering to mitigate vibration-induced noise. Force sensors are embedded at the user-exoskeleton interface, providing stable measurements even under external loads. These sensors are unaffected by sweat and maintain alignment over time due to their structural integration. EMG signals are highly user-specific, non-stationary, and sensitive to electrode placement and skin conditions (changes in electrical impedance) [11,64], which complicates long-term reliability and increases setup time (effort required for threshold tuning) [57,71], making them less practical for industrial applications. Vision-based systems, such as those using depth cameras or IR sensors, are immune to mechanical noise, though they may struggle in dynamic or cluttered environments. Sensor selection should align with application-specific needs, such as precision and robustness. Multimodal approaches that combine biological and non-biological sensors can enhance reliability and adaptability further in industrial settings.

### 5.3. Computation and Models

In line with previous reviews [11,74,91], approaches for predicting user motion or joint torque can be broadly categorized into classification and regression. This two-stage strategy is especially valuable in dynamic environments, where initial classification allows prediction models or control strategies to adapt to task-specific requirements [51,57]. For instance, integrating activity recognition techniques, like those from Pesenti et al. [92], who used LSTM networks to detect lifting actions and payloads, could support context-aware regression models for more precise intent estimation under varying industrial conditions.

The reviewed classification models included threshold-based methods, SVMs, NNs, and recurrent architectures such as LSTMs. These models are typically optimized for prediction accuracy, but their applicability in industrial contexts is constrained by challenges in reliably distinguishing between user states, computational latency, and extensive data requirements. Although SVMs have demonstrated strong performance in online prediction tasks [52,54], the reported computational cost is high, which may hinder deployment in real-time systems. Key design parameters, such as sliding window size and feature selection, significantly influence performance and latency. A critical bottleneck, especially for deep learning-based models, is the limited availability of annotated datasets reflecting realistic industrial scenarios. Only a few multimodal datasets, such as [93,94], closely resemble actual workplace conditions. Broader access to such benchmarks would improve the generalizability and comparability of classification models. Recent research indicates that large language models (LLMs) can help address this gap. For example, LLMs have been used to automatically annotate sensor data streams with semantic labels [95] and enable human-in-the-loop model retraining through natural language interaction [96,97], potentially accelerating adaptation to diverse real-world environments.

Regression models, particularly those based on biomechanical or physical interaction modeling, support continuous estimation of joint torques or motion trajectories and inherently incorporate variations in external dynamics [57,64]. These models provide a detailed and interpretable representation of the support context but require extensive calibration specific to both the user and the device, making them sensitive to deviations from underlying assumptions. As a result, their adaptability to different users, tasks, or deployment scenarios is limited, reducing scalability in diverse industrial settings. In contrast, model-free regression methods offer greater flexibility and can adapt to changing support conditions through retraining. However, they require substantial amounts of training data and careful hyperparameter tuning and often function as black-box models, which can hinder transparency and trust, especially in time-sensitive or safety-critical industrial applications.

Ultimately, the choice between classification, regression, or hybrid approaches depends on the variability and dynamics of the environment and users. Classification excels at detecting discrete actions but depends on accurate labeling and discrete task definitions. Biomechanical regression provides interpretable estimates incorporating external dynamics but requires careful calibration and modeling. Model-free regression offers adaptability but is data-intensive and less transparent, posing challenges for rapid, trustworthy deployment in industrial contexts. Importantly, these methodological trade-offs are closely linked to real-time constraints and the timing of intention prediction, which determine how effectively the system can synchronize with the user’s movements during task execution. Trajectory-following approaches, in particular, must remain strictly aligned with user motions, whereas torque-based methods are generally less time-critical, though too early or late torque onset can reduce support efficiency [57]. Timing aspects depending on used signals and models are examined in detail in Section 5.4.

### 5.4. Time

The timing of intention prediction in upper-limb exoskeletons varies significantly across methods due to differences in underlying mechanisms. Biological signals, such as EMG and EEG, enable early detection of user intent by capturing neuromuscular or cognitive activity before physical movement occurs. However, biological signals are prone to noise and require computational resources for processing, with electromechanical delays further contributing to timing variability [11,68].

Non-biological signals, such as those from IMUs or motion capture systems, rely on observable physical movement, introducing inherent delays. While these methods are more stable and less sensitive to environmental factors, they lack the temporal immediacy of biological signals. For example, IMU-based systems detect motion onset only after a velocity or acceleration threshold is exceeded, resulting in slower response times [18,71].

Computational models also influence prediction timing. Classification models, such as SVMs, are faster but less flexible, while regression models, including deep learning approaches like LSTMs, offer greater adaptability at the cost of additional latency due to algorithmic complexity. Hardware and software configurations, such as sensor sampling rates and data filtering, further impact response times [7,30].

The specific application context also plays a role. Tasks requiring high precision may tolerate longer response times, while rapid or high-force movements demand shorter delays. Subsequent research should focus on optimizing multimodal approaches, improving real-time processing, and tailoring systems to industrial tasks to balance responsiveness and stability [20,68]. Studies should also incorporate user reaction times to a given start signal to relate system response times, as in [75]. Additionally, the system’s total response time should be reported to enhance comparability between different implementations. To capture habitual cues, longer observation windows can provide insights into repetitive behavior in context, which could be used to weight current predictions and translate them into new ones by capturing typical user execution patterns.

### 5.5. Exoskeleton Implementations

Researchers implement intention-based support in industrial exoskeletons either to achieve accurate joint angle trajectory tracking or to provide support torques aligned with the user’s joint torques. Among regression-based trajectory-following approaches, Sedighi et al. uniquely employed a detailed biomechanical model, while most others used interaction-based methods with online-estimated or predefined trajectories targeting fixed or detected points. As noted by Riener and Novak [67], trajectory planning simplifies when the number of start and target points is limited, which motivates approaches like object fixation to infer grasping targets. Eye-tracking techniques show promise in structured environments, such as precision tasks at single workstations (see Table 3), and align with intelligent worker information systems [98] or LLM-based approaches [96]. Scenario classification combined with trajectory generation, as implemented by [50,75], is well suited for repetitive upper-limb movements typical of assembly or lifting activities. Kinematic mechanical models using video tracking [56,61] also rely on calibrated cameras fixed in the workspace and are thus applicable for high-precision, fixed workstation tasks.

For torque prediction, most studies derive support torque from muscle activity measurements. Biomechanical models automatically compensate for varying external loads [64,65] but become complex for multi-degree-of-freedom exoskeletons and require extensive user- and device-specific calibration, often demanding expert knowledge and additional measurement devices. Buongiorno et al. demonstrated that genetic algorithm optimization can automate model adaptation based on user characteristics [53]. Mechanical models have shown robust performance under fixed loads [64] and greater stability against perturbations, critical for safe, real-time control in variable industrial conditions. Such models may be preferred for high-force or load tasks (see Table 3), typically combined with torque or admittance control. Incorporating interaction force measurements or context cues about handled loads can further enhance model performance. While admittance control allows for real-time adjustment, ensuring stability in complex interactions remains challenging [91]. Industrial exoskeletons for shoulder [75] and back support [57] often implement state-machine control triggered by threshold crossings, which can provide reliable support when the thresholds are well defined, for example, in repetivitve tasks, such as assembly lines or lifting activities.

Our review supports prior findings [3,18,99] that evaluation protocols for exoskeleton support and intention models lack standardization, complicating direct comparisons. Most studies are conducted in laboratory settings with non-target users and simulated tasks, limiting their relevance to real industrial environments. Only two studies explicitly evaluated models in industrial tasks [57,75]. Some research includes complex daily activities, such as drinking or object manipulation, which partially resemble industrial motions. In the future, promising methods (see Table 3) could be implemented in active industrial exoskeletons and tested in a multi-stage evaluation process to ensure efficacy and usability in industrial contexts while reducing evaluation efforts (see Section 6).

Beyond the lack of representative evaluation needed to ensure model efficacy and usability, there are additional general barriers to successful exoskeleton implementation in industrial contexts. Selecting appropriate exoskeleton hardware must be done in close alignment with the specific work processes, movement patterns, and the types of tools and loads handled, as highlighted by [100]. Independent of proactive control, mechanical design parameters and the choice of supported joints should be matched to the actual demands of the work process to maximize both support efficiency and user acceptance. Furthermore, expanding the number of well-designed field studies (see Section 6) would not only provide a clearer understanding of real-world efficacy and usability but also help companies to better assess the return on their investment. Such evidence is critical for fostering trust, accelerating adoption, and informing the development of common standards and regulations, a need previously emphasized by [101].

In the context of worker safety, identifying the hazards associated with intention-aware, active, and adaptive industrial exoskeletons requires recognizing risks such as mispredictions, unintended movements, and ergonomic mismatches. Poorly calibrated or ill-fitting systems can lead to strain or injury. Additionally, sensor malfunctions, such as errors in EMG or IMU units, pose safety concerns. An effective risk assessment requires task-specific analysis of activities such as high-force lifting or precision tasks. Furthermore, variability in user anthropometry and potential failure modes, such as power loss, software glitches, and model errors, must be thoroughly evaluated to mitigate risks and enhance safety during deployment. This also includes the impact of exoskeleton-, task-, or human-induced domain shifts on human activity or intention recognition models and methods. Such systems must comply with occupational safety regulations or ISO standards upon deployment. From a workplace perspective, there should be an opt-out culture if discomfort or safety concerns arise.

Recent statistics show no significant decrease in ergonomic risk reduction in industrial and service occupations [102]. Worker safety-II principles [103,104] can be aligned with predictive exoskeletons to reduce risks further. Safety-II is based on the idea that performance adjustment and variability are key factors, and proactive safety management involves making adjustments before negative outcomes occur. Exoskeletons with adjustable support characteristics and multi-modal sensors can respond appropriately to varying tasks and loads, overcoming the bimodality principle in the safety domain and focusing on the presence of positives and how work is really performed (i.e., “Work-As-Done”).

## 6. Future Research and Recommendations

The future development of industrial exoskeleton control should emphasize robustness, adaptability, and user acceptance. Mechanical and model-free approaches using IMUs or force sensors offer high fault tolerance, making them well-suited for industrial settings, though they often react more slowly than EMG-based biomechanical models, which can detect movement prior to its initialization. Integrating contextual knowledge, such as loads, tools, or activities, can improve the support characteristic. To enhance generalizability across domains, transfer learning techniques, including pre-training with motion capture data and adapting to simpler sensor setups, are promising [105]. Furthermore, federated and reinforcement learning allow for on-site model adaptation without centralized data storage. Large language models (LLMs) present a novel opportunity by enabling real-time retraining through user feedback, enabling generalization of pre-trained models.

In industrial environments, sensors must be easy to apply with minimal setup. IMUs, force sensors, or embedded sensors in the exoskeleton are most practical. While EMG sensors are widely used in research, their sensitivity to placement and inter-user variability limits their applicability to industrial contexts. However, recent progress in dry textile EMG sensors suggests that they could be a feasible alternative in the future.

Although most analyzed studies originate from rehabilitation, many of the underlying methods, especially biomechanical and load-based models, are directly applicable to industrial tasks, as they account for external loads that increase joint stress and the risk of musculoskeletal disorders. Recognizing such loads enables one to automatically adjust the support. Before field deployment, these models should be validated in standardized lab tests. Classification-based models can often be reused with adjusted thresholds or retrained on task-specific data. Cognitive signals, such as fatigue or stress, can extend prediction models, enhancing personalization [106]. Long-term monitoring of habitual patterns may also improve intent estimation over time [107,108].

For implementation, we recommend beginning with generalizable task modes, as shown in Table 3. These include force, speed, precision, and varying activity types, each representing common industrial activities. For example, the force mode targets lifting and carrying tasks, speed mode supports rapid motion, precision mode assists fine motor tasks like assembly or welding, and varying mode enables selective support during non-repetitive tasks, such as construction or setup work. The choice between torque- or position-based control should be based on task-specific demands for force and movement.

For reliable evaluation of intention prediction controlled industrial exoskeletons, we propose a three-level evaluation process that integrates technical model performance assessment, preliminary controlled laboratory testing with simulated work tasks, and long-term field trials with the actual target group to capture realistic work patterns and side tasks often absent from laboratory studies [18,109,110,111]. In the first stage, model performance can be evaluated independently of real-world applications using the standard outcome measures identified in this review (see Table A2). For classification, we prefer F1-score over accuracy to account for imbalanced datasets. Metrics should be consistently reported and accompanied by open data to facilitate cross-study comparisons. If model performance meets predefined thresholds, evaluation should proceed to controlled laboratory testing with standardized simulated work tasks, as proposed by [112,113], using established exoskeleton evaluation metrics. Extending prior work on evaluation criteria [99], we recommend measuring the following:

- EMG-based muscle activity of targeted, antagonistic, and non-target muscle groups to assess whole-body load and stress distribution.
- Motion capture-based kinematic changes on whole-body movements analyzed via PCA analysis; see, for example, [114].
- Somatic indicators such as metabolic cost (via respirometry), heart rate, and skin temperature.
- Functional task performance (execution time and execution quality).

All metrics should be benchmarked against both baseline (no exoskeleton) and passive-mode conditions without active support control. Promising configurations should then advance to long-term field trials with end-users. While initial efficacy testing in simulated environments provides valuable technical insights, it lacks demographic representativeness and may not capture real-world usability challenges. To mitigate this, we emphasize the importance of subsequent long-term field studies with actual end-users in their workplace contexts. Although sample sizes may still be limited, these real-world evaluations ensure that the tested populations better reflect the industrial workforce, including a diverse range of ages and gender identities. Given the demographic trends towards an aging workforce, particular care should be taken to include older users in usability assessments to capture age-related ergonomic and acceptance issues. The selection of appropriate exoskeleton hardware should match workplace conditions, following recommendations in [100]. Usability evaluation should begin with a closely monitored one-week familiarization phase, allowing users to adjust mechanical and software settings and address open challenges. This should be followed by a self-managed six- to eight-week structured test phase, during which users provide brief post-use feedback on usage time and usability via short questionnaires focusing on the key factors driving acceptance [16]. The questionnaire should be accessible online or offline and include Likert-scale ratings of perceived work performance, physical strain relief, and wearing comfort. Coupled with usage frequency, duration logs, and structured interviews at the start, mid-point, and end, this approach can yield detailed yet manageable insights into the usability and effectiveness of proactively controlled exoskeletons in real industrial environments. Following this framework can reduce variability in reported metrics and provide a reproducible basis for systematic benchmarking. The staged process also enables early identification of model-related issues before resource-intensive field testing, while ensuring that full software–hardware integration is ultimately validated under real working conditions. To facilitate transparent comparison across studies and accelerate progress in the field, we strongly encourage researchers to publish their datasets along with detailed descriptions of experimental protocols and metrics so that intention prediction models and exoskeleton control strategies can be assessed on common grounds.

## 7. Conclusions

This systematic review, conducted following the PRISMA guidelines, identified 29 studies published between 2007 and 2024 that investigated intention prediction in active exoskeletons for industrial applications. We examined the motivation behind integrating intention prediction and assessed the methods’ relevance for industrial applications. In response to our first research question, *How is intention prediction in upper-limb industrial exoskeletons implemented*? we found that current approaches employ a variety of sensor modalities, most commonly motion capture and electromyography, in combination with model-based and model-free regression and classification approaches. Predictions target joint angles or joint torques, with temporal prediction windows ranging from several hundred milliseconds before motion onset to shortly after movement initiation. Secondly, we asked, *Why is intention prediction in upper-limb industrial exoskeletons implemented?* This review revealed that studies typically aimed to reduce joint loads or enable smooth trajectory following. To evaluate the former, researchers mostly used muscle activation reduction on the targeted muscles as a metric. For trajectory following, most researchers provided results on the deviation between user and exoskeleton joint movement using, for example, root mean square errors. However, evaluations were predominantly performed in controlled laboratory settings with small, non-representative participant groups and heterogeneous performance metrics and evaluation protocols. Building on these findings, we propose targeted recommendations for developing robust, adaptable, and industry-relevant intention prediction strategies based on requirements of the support context and emphasize real-world validation, broader performance metrics, and long-term field studies.

## Figures and Tables

**Table 1:** Research questions on intention prediction in upper-limb exoskeletons, with guiding sub-questions and corresponding analysis dimensions used for systematic categorization and information extraction.

Research Question	Analysis Dimensions
	**(RQ1)** ***How?***
**A. Sensing and Prediction Approach**	
A1. What intention cues are used?	Intention Cue
A2. What types of sensors are employed?	Sensors
A3. What methods are applied to predict intention?	Method
** B. Temporal Context Consideration**	
B1. What temporal aspects are considered?	Timing
B2. How do sensors influence the temporal context?	Sensors
	**(RQ2)** ***Why?***
**C. Purpose and Targeting**	
C1. What is the prediction objective?	Support Characteristic
C2. Who are the target users?	Target Users
** D. Control and Evaluation**	
D1. How is the intention prediction integrated into control?	Support Characteristic
D2. How is the system evaluated?	Evaluation, Target Users

**Table 2:** Included publications and their contribution to the dimensions of analysis.

Reference	Intention Cue	Sensors	Method	Timing	Support Charac.	Target Users	Evaluation
Bandara et al. [52]	✓	✓	✓	✓	-	-	-
Bi et al. [11]	✓	✓	✓	-	-	-	✓
Buongiorno et al. [53]	-	✓	✓	-	✓	✓	-
Chen et al. [54]	✓	✓	✓	✓	-	-	-
Dinh et al. [7]	-	✓	✓	✓	✓	✓	✓
Gandolla et al. [55]	✓	✓	✓	-	✓	✓	✓
Gantenbein et al. [18]	✓	✓	-	-	-	-	✓
Gao et al. [56]	-	✓	✓	-	✓	✓	✓
Heo et al. [57]	✓	✓	✓	✓	✓	✓	✓
Huo et al. [58]	-	✓	✓	-	✓	✓	✓
Irastorza-Landa et al. [59]	✓	✓	✓	✓	-	-	-
Khan et al. [60]	-	✓	✓	-	✓	✓	✓
Liao et al. [61]	-	✓	✓	✓	✓	✓	✓
Losey et al. [62]	✓	✓	✓	✓	-	-	-
Lotti et al., 2020 [63]	✓	-	✓	✓	✓	✓	✓
Lotti et al., 2022 [64]	✓	✓	✓	✓	✓	✓	✓
Lu et al. [65]	-	✓	✓	-	✓	✓	✓
Novak and Riener [66]	✓	✓	✓	✓	✓	✓	✓
Riener and Novak [67]	✓	✓	✓	✓	✓	✓	✓
Sedighi et al. [68]	✓	✓	✓	✓	✓	✓	-
Sun et al. [69]	✓	-	✓	✓	-	-	✓
Toro-Ossaba et al. [70]	✓	✓	✓	✓	✓	✓	-
Treussart et al. [71]	✓	✓	✓	-	✓	✓	-
Woo et al. [72]	✓	✓	✓	-	-	-	-
Zabaleta et al. [50]	✓	✓	✓	-	✓	✓	✓
Zarrin et al. [73]	✓	-	✓	-	✓	✓	✓
Zhang et al., 2019 [74]	✓	✓	✓	-	-	-	-
Zhang et al., 2023 [51]	-	✓	✓	✓	✓	✓	✓
Zhou et al. [75]	✓	-	✓	✓	✓	✓	✓

**Table 3:** Recommendations for the development of intent-aware exoskeletons for specific fields of application. We use four classes of activities: force, varying, speed, and precision activities, which have specific requirements on torque or motion accuracy.

	Activity Type	Description	Examples	Signal, Sensor	Control	References
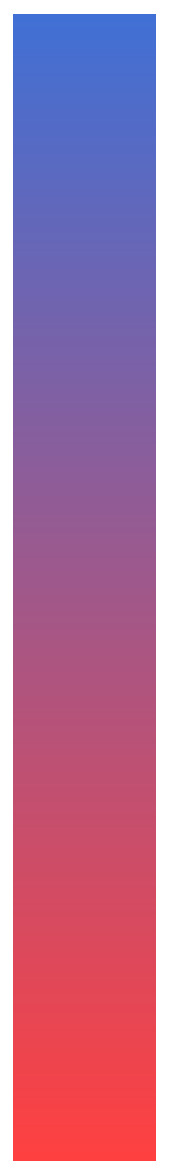	**Force**	high weights, low velocity, small motion range	lifting objects, heavy tool handling, hammering	kinematic, mechanical cues IMUs, embedded motor sensors, force/torque sensors	admittance control, torque control	[7,53,58,63,71]
	**Varying**	different weights, different velocities, small motion range	construction work, commissioning, workplace setup	kinematic, mechanical cues IMUs, embedded motor sensors, force/torque sensors, context information	torque control, Model Reference Adaptive control	[57,67,70,71,75]
	**Speed**	low-middle weights, high velocity, high motion range	object sorting, transporting, drilling, cleaning	kinematic cues IMUs, embedded motor sensors	position control	[51,55,68,73]
	**Precision**	low weights, low velocity, small motion range	small part assembly, painting, welding	kinematic cues IMUs, embedded motor sensors, camera	admittance control, position control	[55,58,60,61,68,73]
		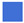 High Torque Accuracy 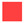 High Motion Accuracy				

## Data Availability

The data presented in this study are available upon request from the lead author.

## Supplementary Materials

The following supporting information can be downloaded at https://www.mdpi.com/article/10.3390/s25175225/s1. File S1: PRISMA Process Data; File S2: PRISMA Checklist; File S3: PRISMA Abstract Checklist.

## Appendix A. Detailed Results

### Appendix A.1. Biological Signals

**Table A1:** Placement of gel-based sEMG control sensors, grouped by estimation objectives and limb region.

		Elbow Torque@					Elbow Shoulder Torque	Elbow Shoulder Angle	Motion Classification					
	Muscle	[70]	[64]	[63]	[65]	[7]	[53]	[68]	[75]	[59]	[54]	[57]	[67]	[50]
**Upper Limb**	Biceps Brachii	✓	✓	✓	✓	✓	✓	✓	✓	✓	✓		✓	
	Triceps Brachii	✓	✓	✓			✓			✓	✓			
	Brachioradialis			✓										
	Anterior Deltoid						✓	✓	✓	✓	✓		✓	
	Posterior Deltoid						✓			✓	✓		✓	
	Medial Deltoid							✓	✓		✓		✓	
	Lateral Deltoid									✓				
	Supraspinatus										✓			
	Infraspinatus										✓			
	Teres Major										✓			
	Pectoralis Major										✓			
	Trapezius										✓		✓	
	Wrist Flexor										✓			
	Wrist Extensor										✓			
**Lower Limb**	Gluteus Maximus											✓		✓
	Biceps Femoris											✓		
	Vastus Lateralis													✓

### Appendix A.2. Users and Evaluation

**Table A2:** Summary of model performances, categorized by classification and regression approaches. Models are grouped by type and compared using reported evaluation metrics. Only studies with quantitative results are included; if multiple models were reported, only the best-performing one is shown. (RMSE = root mean square error; R^2^ = coefficient of determination).

	Ref.	Model Type	Evaluation Metric	Result
**Classification**	[52]	SVM; activity classification	accuracy; offline	73.1%
	[54]	SVM; activity classification	accuracy; offline	90–98%
			accuracy; online	81–99%
	[59]	SVM; movement intention	accuracy	93.03%
		SVM; movement direction	accuracy	78.28%
	[51]	Random Forest	accuracy	97.0±0.9% no support; 93.0±3.1% support
	[55]	thresholding; kinematics-based	accuracy	99%
	[57]	thresholding; kinematics-based	accuracy	92.6%
		thresholding; EMG-based	accuracy	96.2%
	[67]	thresholding; EMG/force-based	false positives; online	3.9%
	[50]	thresholding; kinematics/force-based	accuracy	100% unimpaired; 0% impaired
	[75]	thresholding; kinematics-based	accuracy	98.7–99.7%
		thresholding; EMG-based	accuracy	57.4–95.6%
**Regression**	[53]	biomechanical model	R^2^	0.90±0.04 elbow 0.92±0.03 shoulder
			RMSE	1.39±0.25Nm elbow 1.73±0.36Nm shoulder
	[63]	biomechanical model	R^2^	0.87±0.04
			RMSE	0.01±0.005Nm/kg
	[65]	biomechanical model	correlation coefficient	98.04%
			RMSE	1.90 Nm
	[71]	biomechanical model	RMSE	0.04±0.01Nm
	[73]	mechanical geodesics model	RMSE	0.02–0.13 mm
	[51]	Random Forest	R^2^	0.90±0.02
			RMSE	$0.13\pm 0.01\;\mathrm{rad}/s^{-1}$
	[60]	Extreme Learning Machine	RMSE	4.2±6°
	[68]	hybrid CNN-LSTM model	accuracy	91.2%
	[69]	NARX neural network	RMSE	0.87–1.1°
	[70]	multilayer neural network	mean absolute error	0.58 at 0 kg; 1.53 at 5 lb
